# Opium Habit and Amyl Nitrite

**Published:** 1879-03

**Authors:** 


OPIUM HABIT AND AMYL NITRITE.—Dr. Leyman (*Boston Med. and Surg. Jour.*) has successfully used *amyl nitrite* in insomnia consequent upon suddenly discontinuing the opium habit. Two or three whiffs, the *flushing of the face being the criterion*, were usually sufficient, being followed by refreshing sleep.

